# Fatherhood status and risk of prostate cancer: Nationwide, population-based case–control study

**DOI:** 10.1002/ijc.28057

**Published:** 2013-03-08

**Authors:** Sara M Wirén, Linda I Drevin, Sigrid V Carlsson, Olof Akre, Erik C Holmberg, David E Robinson, Hans G Garmo, Pär E Stattin

**Affiliations:** 1 Department of Surgery and Perioperative Sciences, Urology and Andrology, Umeå UniversityUmeå, Sweden; 2 Regional Cancer Centre, Uppsala University HospitalUppsala, Sweden; 3 Department of Urology, Sahlgrenska Academy, University of GothenburgGothenburg, Sweden; 4 Department of Surgery, Urology Service, Memorial Sloan-Kettering Cancer CenterNew York, NY; 5 Clinical Epidemiology Unit, Department of Medicine Solna, Karolinska InstitutetStockholm, Sweden; 6 Regional Cancer Centre, Sahlgrenska University HospitalGothenburg, Sweden; 7 Department of Oncology, Institute of Clinical Sciences, Sahlgrenska Academy, University of GothenburgGothenburg, Sweden; 8 Department of Urology, Ryhov County HospitalJönköping, Sweden; 9 King's College London, School of Medicine, Division of Cancer Studies, Cancer Epidemiology GroupLondon, United Kingdom

**Keywords:** prostate cancer, epidemiology, case–control studies, hypogonadism, androgens

## Abstract

Previous studies have shown a decreased risk of prostate cancer for childless men; however, the cause of the association remains to be elucidated. The aim of our study was to assess the risk of prostate cancer by fatherhood status, also considering potential confounding factors. In a case–control study in Prostate Cancer data Base Sweden 2.0, a nationwide, population-based cohort, data on number of children, marital status, education, comorbidity and tumor characteristics obtained through nationwide healthcare registers and demographic databases for 117,328 prostate cancer cases and 562,644 controls, matched on birth year and county of residence, were analyzed. Conditional logistic regression was used to estimate odds ratios (ORs) and 95% confidence intervals (95% CIs) for prostate cancer overall and by risk category, adjusting for marital status and education. Childless men had a decreased risk of prostate cancer compared to fathers, OR = 0.83 (95% CI = 0.82–0.84), and risk was lower for low-risk prostate cancer, OR = 0.74 (95% CI = 0.72–0.77), than for metastatic prostate cancer, OR = 0.93 (95% CI = 0.90–0.97). Adjustment for marital status and education attenuated the association in the low-risk category, adjusted OR = 0.87 (95% CI = 0.84–0.91), whereas OR for metastatic cancer remained virtually unchanged, adjusted OR = 0.92 (95% CI = 0.88–0.96). Our data indicate that the association between fatherhood status and prostate cancer to a large part is due to socioeconomic factors influencing healthcare-seeking behavior including testing of prostate-specific antigen levels.

Previous studies on the association between fatherhood status and risk of prostate cancer have yielded inconsistent results.^1^–^9^ Two large Scandinavian register-based studies reported a decreased prostate cancer risk among childless men compared to fathers.^2^,^6^

It has been proposed that the cause of the association between fatherhood status and risk of prostate cancer is a difference in androgenicity.^2^ Androgens are necessary for prostate and prostate cancer development, and intraprostatic androgen levels have been implicated to affect the risk of prostate cancer.^10^–^12^ Infertile men on average have lower serum levels of androgens than fertile men.^13^,^14^ Thus, under the assumption that some men are childless due to male infertility, being childless may be a proxy for long-term low androgen exposure. However, there are many reasons for childlessness besides infertility, such as a lack of female partner, female infertility or no desire to have children.^15^

Childless men may also differ from fathers with respect to general health, socioeconomic factors, such as marital status, education and income and healthcare-seeking pattern, including uptake of serum prostate-specific antigen (PSA) testing, factors that are all related to the risk of prostate cancer diagnosis and such factors may confound an association. None of the previous studies on the association between fatherhood status and risk of prostate cancer accounted for all these potential confounders.^1^–^3^,^6^,^7^

We conducted a case–control study of the association between fatherhood status and risk of prostate cancer in Prostate Cancer data Base Sweden (PCBaSe) 2.0, including more than 117,000 cases of prostate cancer, taking into account marital status, education, comorbidity and tumor characteristics.

## Material and Methods

### The National Prostate Cancer Register of Sweden

In 1987, the first regional prostate cancer register was established in south-east Sweden and subsequentially more regions joined to form the National Prostate Cancer Register (NPCR) of Sweden.^16^ Since 1998, NPCR is nationwide, and the capture rate is more than 97% in comparison to the Swedish Cancer Register to which registration is mandatory and is regulated by law. NPCR contains information on diagnostic unit, date of diagnosis, tumor characteristics according to the tumor, node, metastasis classification, data on tumor differentiation, serum level of PSA at diagnosis and primary treatment delivered or decided within 6 months after diagnosis. Data on cause for diagnostic work-up leading to the prostate cancer diagnosis are available from 2000 and onward.

### PCBaSe 2.0

PCBaSe was created in 2008, and it is based on linkages between the NPCR and a number of other nationwide demographic and health registers.^17^ Linkage was performed using the unique personal identity number assigned to each Swedish resident.^18^ In 2011, a new linkage with more cases of prostate cancer and longer follow-up was performed and PCBaSe 2.0 was created.^19^ By using the data from the NPCR, the Swedish Multi-Generation Register, the Longitudinal Integration Database for Health Insurance and Labor Market Studies (LISA) and the National Patient Register, information was obtained on tumor characteristics, number of children, educational level and comorbidity. No formal screening for prostate cancer has been or is currently in operation in Sweden apart from in the Gothenburg area where 10,000 men have been invited to PSA screening as part of a randomized trial.^20^

### Identification and categorization of cases

We included all cases of prostate cancer in PCBaSe 2.0 diagnosed between 1991 and 2009 in our analysis and classified them into five risk categories according to a modification of the National Comprehensive Cancer Network^21^: low-risk: local clinical stage T1–2, Gleason score = ≤6 and serum levels of PSA < 10 ng/mL; intermediate-risk: T1–2, Gleason score = 7 and/or PSA 10 to <20 ng/mL; high-risk: T3, Gleason score = 8–10 and/or PSA 20 to <50 ng/mL; regionally metastatic disease: T4 and/or N1 and/or PSA 50 to <100 ng/mL in the absence of distant metastases (M0 or Mx) and distant metastases: M1 and/or PSA ≥ 100 ng/mL.

### Control sampling

For each case of prostate cancer, two controls for the period of 1991–1995 and five controls for the period of 1996–2009 were randomly sampled among prostate cancer free men in the Swedish population matched for birth year (±1 year) and county of residence.

### Identification of children

We ascertained fatherhood status for cases and controls by using the Multi-Generation Register, a nationwide register created in March 2000 and kept at Statistics Sweden. It includes all subjects born 1932 or later who were alive in 1961.^22^ For each index subject, the parents and thus all first-degree relatives can be identified. Overall, there is virtually complete parental information on the index subjects, but for those who died before 1991; the completeness is ∼50%. Adoptions and other nonbiological relationships are flagged and were not included in the current analysis.

### Assessment of comorbidity

The Patient Register includes all diagnosis from in-patient hospital care in Sweden since 1987.^23^ The register is kept at the National Board of Health and Welfare. Since 1985, the proportion of missing personal identity numbers has been less than 1%, and since 1964, the proportion of missing data on main diagnosis has been 1%. By using the discharge diagnoses in the Patient Register for the 10 years preceding the date of diagnosis of prostate cancer, we classified cases and controls into four categories of comorbidity using Charlson comorbidity index: 0, 1, 2, 3+ comorbidities.^24^

### Assessment of marital status and educational level

We used the LISA, a nation-wide demographic database kept at Statistics Sweden that includes all subjects aged above 16 years residing in Sweden, to obtain information on marital status for the same year as the date of diagnosis of the case. Marital status was classified into married (also including registered partnership), divorced, widower and never married. LISA was also used to obtain information on the highest attained educational level, and this information was available for the period of 1990–2009. The highest level of education was recorded the same year as diagnosis of prostate cancer in cases (or in the index case for the controls). If information was not available for that year, the latest recorded educational level was used. We then categorized educational level in four categories: low (9-year grade school), intermediate (high school), high (college) and other.

### Statistical analysis

Odds ratios (ORs), with 95% confidence intervals (95% CIs), for the risk of prostate cancer by fatherhood status, marital status, education and comorbidity index were calculated using conditional logistic regression.^25^ In stratified analysis, we also evaluated whether the associations were different in the five prostate cancer risk categories as well as in the categories of mode of detection of prostate cancer, marital status, education and comorbidity. The ORs were adjusted for education and marital status as these factors were associated with the risk of prostate cancer in our analyses. All statistical tests were two-sided, and the *α*-level was 0.05. All tests were performed using the R statistical program package (2.12.0).^26^ Our study was approved by the Research Ethics Board of Umeå University Hospital.

## Results

Of the 117,328 cases and 562,644 controls, a higher proportion of cases, 84.1%, compared to controls, 81.0%, were fathers (Table 1). A higher proportion of cases, 68.0%, compared to controls, 64.3%, were married, and slightly more cases, 51.5%, compared to controls, 49.4%, had an intermediate or high educational level.

**Table 1:** Characteristics of cases and controls in PCBaSe 2.0

	Cases (*n* = 117,328) *n* (%)	Controls (*n* = 562 644) *n* (%)
**Age (years)**		
<65	27,724 (23.6)	136,029 (24.2)
65 to <75	42,988 (36.6)	206,317 (36.7)
≥75	46,616 (39.7)	220,298 (39.2)
**Fatherhood status**		
Childless	18,689 (15.9)	106,700 (19.0)
Father of one or more children	98,639 (84.1)	455,944 (81.0)
**Marital status**^1^		
Married	79,735 (68.0)	361,825 (64.3)
Divorced	13,870 (11.8)	72,935 (13.0)
Widower	13,284 (11.3)	63,371 (11.3)
Never married	10,428 (8.9)	64,513 (11.5)
Missing data	11 (0.0)	0 (0.0)
**Educational level**^2^		
Low	52,883 (45.1)	264,844 (47.1)
Intermediate	39,145 (33.4)	184,456 (32.8)
High	21,345 (18.2)	93,757 (16.7)
Other	3,955 (3.4)	19,587 (3.5)
**Comorbidity**^3^		
0	76,129 (64.9)	362,192 (64.4)
1	21,466 (18.3)	102,674 (18.2)
2	11,515 (9.8)	54,973 (9.8)
>3	8,218 (7.0)	42,805 (7.6)

1 Marital status was determined the same year as diagnosis of prostate cancer in cases (or in the index case of the controls).

2 Highest level of education was recorded the same year as diagnosis of prostate cancer (or in the index case for the controls) or the latest recorded educational level.

3 Classified according to Charlson comorbidity index.^24^

A much higher proportion of fathers, 71.6%, than childless men, 35.3%, were married, and a higher proportion of fathers, 52.1%, than childless men, 39.7%, had an intermediate or high educational level (Table 2). A higher proportion of fathers, 22.1%, than childless men, 17.0%, were diagnosed with prostate cancer as a result of a health examination (*i.e*., PSA testing). Almost half of the cases were diagnosed during work-up of symptoms (Table 3).

**Table 2:** Characteristics of fathers and childless men in PCBaSe 2.0

	Fathers (*n* = 554,583) *n* (%)	Childless men (*n* = 125,389)*n* (%)
**Marital status**^1^		
Married	397,293 (71.6)	44,267 (35.3)
Divorced	77,724 (14.0)	9,081 (7.2)
Widower	64,331 (11.6)	12,324 (9.8)
Never married	15,228 (2.7)	59,713 (47.6)
Missing data	7 (0.0)	4 (0.0)
**Educational level**^2^		
Low	251,616 (45.4)	66,111 (52.7)
Intermediate	189,343 (34.1)	34,258 (27.3)
High	99,560 (18.0)	15,542 (12.4)
Other	14,064 (2.5)	9,478 (7.6)
**Comorbidity**^3^		
0	357,270 (64.4)	81,051 (64.6)
1	101,524 (18.3)	22,616 (18.0)
2	54,244 (9.8)	12,244 (9.8)
3+	41,545 (7.5)	9,478 (7.6)
**Prostate cancer cases**		
Mode of detection^4^		
Health examination	21,765 (22.1)	3,182 (17.0)
Symptoms	45,780 (46.4)	8,895 (47.6)
Other	5,503 (5.6)	999 (5.3)
Missing data	25,591 (25.9)	5,613 (30.0)

1 Marital status was determined the same year as diagnosis of prostate cancer in cases (or in the index case of the controls).

2 Highest level of education was recorded the same year as diagnosis of prostate cancer (or in the index case for the controls) or the latest recorded educational level.

3 Classified according to Charlson comorbidity index.^24^

4 Information on diagnostic work-up was recorded in NPCR from 2000.

**Table 3:** Demographics and tumor characteristics of prostate cancer cases in PCBaSe 2.0

	*n* (%)	
**Year of diagnosis**		
1991–1995	7,995 (6.8)	
1996–1999	21,759 (18.5)	
2000–2002	22,305 (19.0)	
2003–2006	37,406 (31.9)	
2007–2009	27,863 (23.7)	
**Mode of detection**^1^		
Health examination	24,947 (21.3)	
Symptoms	54,675 (46.6)	
Other	6,502 (5.5)	
Missing data	31,204 (26.6)	
**Risk category**^2^		Age (mean)
Low-risk	26,402 (22.5)	65.9
Intermediate-risk	26,611 (22.7)	69.4
High-risk	30,159 (25.7)	73.7
Regionally metastatic	10,315 (8.8)	74.2
Distant metastases	20,391 (17.4)	74.8
Missing data	3,450 (2.9)	

1 Information on diagnostic work-up was recorded in NPCR from year 2000.

2 Low-risk: T1–2, Gleason score = 6 and PSA < 10 ng/mL. Intermediate-risk: T1–2, Gleason score = 7 and/or PSA 10 to <20 ng/mL. High-risk: T3, Gleason score = 8–10 and/or PSA 20 to <50 ng/mL. Regionally metastatic disease: T4 and/or N1 and/or PSA 50 to <100 ng/mL in the absence of distant metastases (M0 or Mx). Distant metastases: M1 and/or PSA ≥ 100 ng/mL.

### Association between fatherhood status and risk of prostate cancer

**Figure 1:**
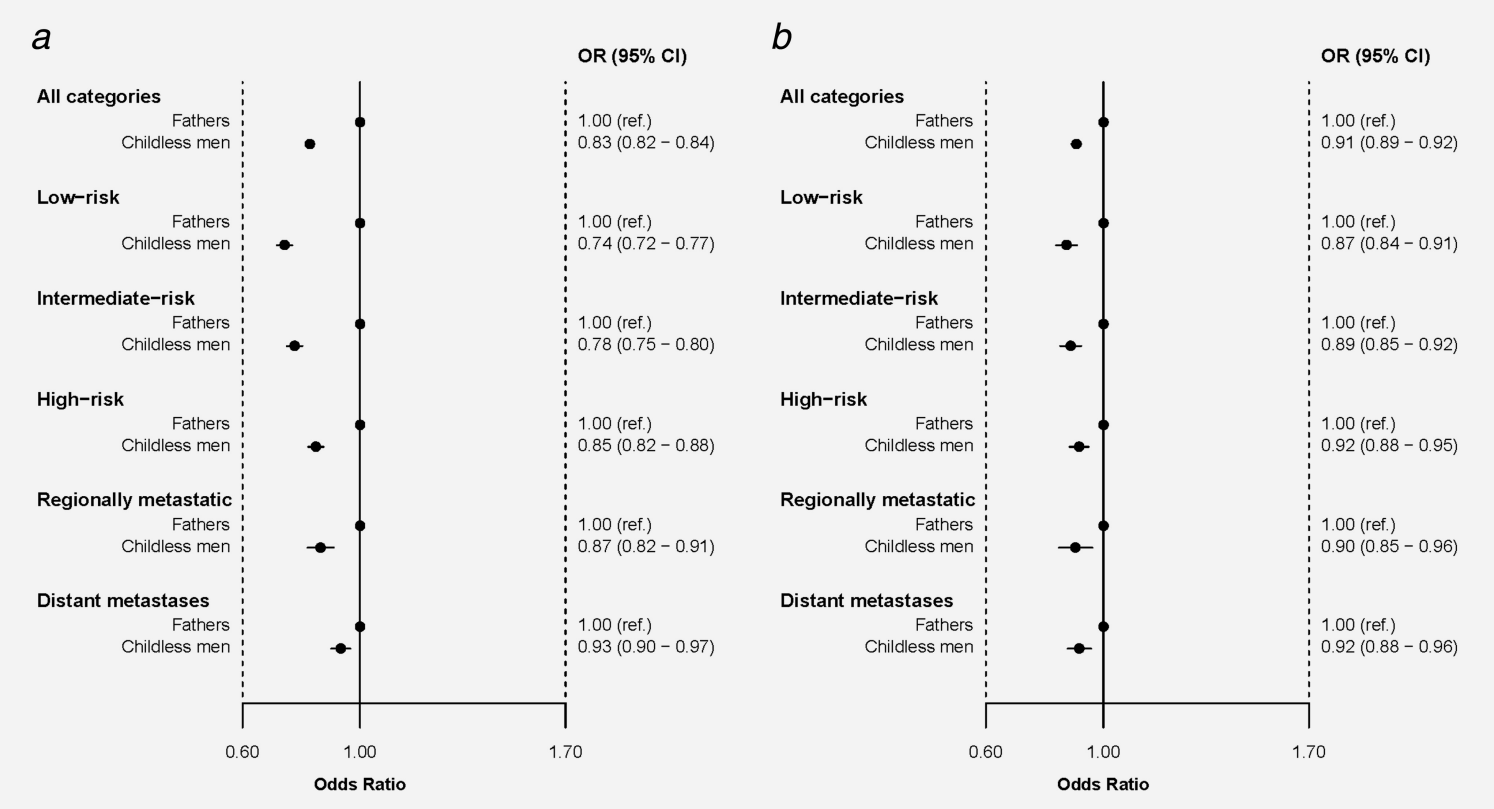
(*a*) Unadjusted risk of prostate cancer by fatherhood status. (*b*) Risk of prostate cancer by fatherhood status, adjusted for socioeconomic status and marital status.

In univariate analysis, childless men had a decreased risk of prostate cancer overall compared to fathers, OR = 0.83 (95% CI = 0.82–0.84), and risk was lower for low-risk prostate cancer, OR = 0.74 (95% CI = 0.72–0.77), than for metastatic prostate cancer, OR = 0.93 (95% CI = 0.90–0.97; Fig. 1*a*).

In multivariate analyses, inclusion of marital status and educational level attenuated the decreased prostate cancer risk for childless men; however, the decreased risk remained statistically significant in all risk categories (Fig. 1*b*). The strongest attenuation was observed in the low-risk group, and the unadjusted OR for childless men compared to fathers was OR = 0.74 (95% CI = 0.72–0.77) and adjusted OR = 0.87 (95% CI = 0.84–0.91), whereas the risk for metastatic disease remained virtually unchanged, OR = 0.93 (95% CI = 0.90–0.97) *versus* OR = 0.92 (95% CI = 0.88–0.96).

### Association between marital status, education, comorbidity and risk of prostate cancer

**Figure 2:**
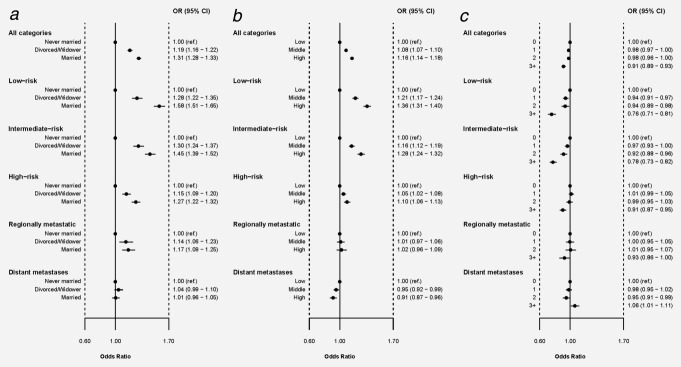
(*a*) Risk of prostate cancer by marital status. (*b*) Risk of prostate cancer by educational level. (*c*) Risk of prostate cancer by Charlson comorbidity index.

Married and divorced men were at increased risk of prostate cancer compared to unmarried men; unadjusted OR = 1.31 (95% CI = 1.28–1.33) for married men and OR = 1.19 (95% CI = 1.16–1.22) for divorced men (Fig. 2*a*). Men with a high educational level had an increased risk of prostate cancer compared to men with a low educational level, OR = 1.16 (95% CI = 1.14–1.18; Fig. 2*b*). The associations for married men were strongest for low-risk and intermediate-risk tumors, whereas there was no association to metastatic disease. The associations for educational level were also stronger for low-risk tumors, and there was a weak inverse association between high educational level and risk of metastatic disease. Comorbidity was not associated to an increased prostate cancer risk overall and was not included in further analyses (Fig. 2*c*).

The association between fatherhood status and prostate cancer was strongest among men diagnosed as a result of PSA testing. For cancers diagnosed by PSA testing, OR was 0.71 (95% CI = 0.68–0.74) for childless men compared to fathers, whereas OR was 0.86 (95% CI = 0.83–0.88) for prostate cancer detected due to symptoms from the lower urinary tract. After adjustment for marital status and education, these estimates were attenuated, and adjusted OR for cancer detected due to PSA testing was 0.86 (95% CI = 0.82–0.89), and for cancer detected due to symptoms, OR was 0.90 (95% CI = 0.87–0.93).

In separate risk analysis in strata of educational level and marital status, the association between fatherhood status and risk of prostate cancer was stronger for low-risk tumors among men with a low educational level, OR = 0.72 (95% CI = 0.68–0.76), than among men with a high educational level, OR = 0.82 (95% CI = 0.76–0.89).

## Discussion

In this large, nationwide, population-based case–control study on fatherhood status and risk of prostate cancer, we found an almost 20% decrease in the risk of prostate cancer for childless men. The decrease in risk was largest for low-risk prostate cancer, and adjustment for educational level and marital status strongly attenuated this estimate. There was a weaker albeit statistically significant decrease in the risk of metastatic cancer that was unaffected by such adjustments. Our data indicate that the association between fatherhood status and prostate cancer to a large part is due to socioeconomic factors influencing healthcare-seeking behavior including testing of PSA levels and that the remaining association may be due to residual confounding as well as to unmeasured confounders.

Four previous studies and one meta-analysis have investigated the association between fatherhood status and prostate cancer risk.^1^–^3^,^6^,^7^ The largest of these studies, a population-based case–control study from Sweden including 48,850 prostate cancer cases reported a 17% lower risk of prostate cancer among men with no or one child compared to men with more than one child.^2^ Accordingly, a 16% lower risk of prostate cancer for childless men compared to men with one or more children was found in a population-based Danish cohort study on 3,400 prostate cancer cases.^6^ In contrast, in a meta-analysis of seven population-based and 10 hospital-based case–control studies, one cohort study of 4,240 cases of prostate cancer and 13,322 controls,^1^ one hospital-based study on 1,294 cases^3^ and one population-based cohort study on 8,134 cases,^7^ no overall association was reported between number of children and risk of prostate cancer.

Lower androgenicity in infertile men compared to fertile men has been implicated as the biological link between fatherhood status and risk of prostate cancer.^2^ The largest study on serum levels of androgens in infertile men, based on 357 men with idiopathic infertility and 318 fertile men, reported that serum testosterone levels were 18% lower in infertile men and that 12% of the infertile men had testosterone levels below the 2.5 percentile of levels in the fertile men.^14^ Low circulating levels of testosterone have not been associated with a decreased risk of prostate cancer in large observational studies^27^; however, lowering levels of dihydrotestosterone, the most potent androgen in the prostate, by the use of 5α-reductase inhibitors for 2–7 years reduced the risk of prostate cancer at biopsy by 25% in two large randomized clinical trials.^28^,^29^

However, studies on testicular function with fatherhood status as a proxy for fertility status are hampered by the fact that fatherhood status is influenced by many factors besides the fertility of the man such as no female partner, fertility of the partner and the desire or ability to have children.^15^ Around 3–4% of couples remain involuntarily childless throughout life, and given that male infertility is a contributing factor in 50% of infertile couples,^30^ we estimated that about 10% of the childless men in our study were infertile. Thus, only a small minority of the childless men in our study can be assumed to have been infertile and have had concomitant low androgen levels. Assuming causality between androgen levels and prostate cancer risk, the undiluted association between low androgen levels heralded by infertility and decreased risk of prostate cancer would have to be substantially stronger than what we observed. However, it is unlikely that this risk would be so strong that it would fully explain the difference in the adjusted risk estimates. Therefore, there are reasons to believe that a large part of the observed association was due to residual confounding.

Two studies have examined the risk of prostate cancer in infertile men with conflicting results.^8^,^9^ A Swedish study, based on self-reported life-long failure to conceive included 445 cases of prostate cancer of which 16 men were infertile, reported that infertile men had half the risk of prostate cancer compared to fertile men.^8^ A US study, based on men seeking medical care for failure to conceive included 168 cases of prostate cancer of which 56 cases had male factor infertility, reported that infertile men had no increase in risk of all or low-grade prostate cancer but a twofold increase in risk of high-grade cancer (Gleason score = 8–10).^9^ This estimate was based on only 19 cases of high-grade cancer with male factor infertility.

Our study shows that educational level and marital status affected the risk of prostate cancer. Married and divorced men had an increased risk of prostate cancer compared to men who had never been married, and men with a high educational level also had an increased risk in accordance with previous studies.^31^–^33^ The increase in risk of prostate cancer was strongest for low-risk disease among married men and men with high education, whereas there was no association between marital status and metastatic disease and there was a weak inverse association between high educational level and risk of metastatic disease. These associations are likely due to a higher uptake of PSA testing among married men and men with high education, and accordingly we found a suggestion of different uptake of PSA testing according to fatherhood status, 17% of childless men were diagnosed with prostate cancer as a result of PSA testing, whereas the corresponding percentage for fathers was 22%. The distribution of comorbidities was similar between the groups, indicating that there were no major differences in general health between the groups. Thus, the decreased risk of prostate cancer observed in childless men in our and previous studies appears to mainly reflect differences in healthcare-seeking patterns between childless men and fathers. However, there remained a modest, albeit statistically significant decrease in the risk of metastatic prostate cancer among childless men even after adjustment for marital status and education. To what extent this association is caused by residual confounding and unmeasured confounding cannot be elucidated using our data.

## Conclusion

Our results indicate that the decreased risk of prostate cancer among childless men to a large part is due to differences in marital status and educational level that both influence healthcare-seeking behavior including testing of PSA levels.

## What's new?

Previous studies have indicated that childless men may have a decreased risk of prostate cancer compared with men who are fathers, possibly because childless men may be infertile and therefore have reduced androgen levels that would otherwise predispose them to disease. This population-based case-control study supports the finding that childless men have a decreased risk of prostate cancer, but adjustment for socioeconomic factors substantially weakened an association for low-risk cancers. The results indicate that the relationship between fatherhood status and prostate cancer may be due largely to socioeconomic factors that influence health-care seeking behavior and hence testing of prostate specific antigen levels.

## References

[1] Dennis LK, Dawson DV (2002). Meta-analysis of measures of sexual activity and prostate cancer. Epidemiology.

[2] Giwercman A, Richiardi L, Kaijser M (2005). Reduced risk of prostate cancer in men who are childless as compared to those who have fathered a child: a population based case-control study. Int J Cancer.

[3] Negri E, Talamini R, Bosetti C (2006). Risk of prostate cancer in men who are childless. Int J Cancer.

[4] Harlap S, Paltiel O, Friedlander Y (2007). Prostate cancer in fathers with fewer male offspring: the Jerusalem Perinatal Study Cohort. J Natl Cancer Inst.

[5] Bermejo JL, Sundquist J, Hemminki K (2007). Re: Prostate cancer in fathers with fewer male offspring: the Jerusalem Perinatal Study Cohort. J Natl Cancer Inst.

[6] Jorgensen KT, Pedersen BV, Johansen C (2008). Fatherhood status and prostate cancer risk. Cancer.

[7] Eisenberg ML, Park Y, Brinton LA (2010). Fatherhood and incident prostate cancer in a prospective US cohort. Int J Epidemiol.

[8] Ruhayel Y, Giwercman A, Ulmert D (2010). Male infertility and prostate cancer risk: a nested case-control study. Cancer Causes Control.

[9] Walsh TJ, Schembri M, Turek PJ (2010). Increased risk of high-grade prostate cancer among infertile men. Cancer.

[10] Hsing AW, Chu LW, Stanczyk FZ (2008). Androgen and prostate cancer: is the hypothesis dead?. Cancer Epidemiol Biomarkers Prev.

[11] Wiren S, Stattin P (2008). Androgens and prostate cancer risk. Best Pract Res Clin Endocrinol Metab.

[12] Crawford ED (2009). Understanding the epidemiology, natural history and key pathways involved in prostate cancer. Urology.

[13] Dohle GR, Smit M, Weber RF (2003). Androgens and male fertility. World J Urol.

[14] Andersson AM, Jorgensen N, Frydelund-Larsen L (2004). Impaired Leydig cell function in infertile men: a study of 357 idiopathic infertile men and 318 proven fertile controls. J Clin Endocrinol Metab.

[15] ESHRE-Workshop (2001). Social determinants of human reproduction. Hum Reprod.

[16] Adolfsson J, Garmo H, Varenhorst E (2007). Clinical characteristics and primary treatment of prostate cancer in Sweden between 1996 and 2005. Scand J Urol Nephrol.

[17] Hagel E, Garmo H, Bill-Axelson A (2009). PCBaSe Sweden: a register-based resource for prostate cancer research. Scand J Urol Nephrol.

[18] Ludvigsson JF, Otterblad-Olausson P, Pettersson BU (2009). The Swedish personal identity number: possibilities and pitfalls in healthcare and medical research. Eur J Epidemiol.

[19] Van Hemelrijck M, Wigertz A, Sandin F (2012). Cohort profile: the National Prostate Cancer Register of Sweden and Prostate Cancer data Base Sweden 2.0. Int J Epidemiol.

[20] Hugosson J, Carlsson S, Aus G (2010). Mortality results from the Goteborg randomised population-based prostate-cancer screening trial. Lancet Oncol.

[21] Mohler J, Bahnson RR, Armstrong AJ (2011). NCCN clinical practice guidelines in oncology: prostate cancer. J Natl Compr Canc Netw.

[22] Ekbom A (2011). The Swedish Multi-Generation Register. Methods Mol Biol.

[23] Ludvigsson JF, Andersson E, Ekbom A (2011). External review and validation of the Swedish national inpatient register. BMC Public Health.

[24] Charlson ME, Pompei P, Ales KL (1987). A new method of classifying prognostic comorbidity in longitudinal studies: development and validation. J Chronic Dis.

[25] Cox DR, Oakes D (1984). Analysis of survival data.

[26] Ihaka R, Gentleman R (1996). R: a language for data analysis and graphics. J Comput Graph Stat.

[27] Roddam AW, Allen NE, Appleby P (2008). Endogenous sex hormones and prostate cancer: a collaborative analysis of 18 prospective studies. J Natl Cancer Inst.

[28] Thompson IM, Goodman PJ, Tangen CM (2003). The influence of finasteride on the development of prostate cancer. N Engl J Med.

[29] Andriole GL, Bostwick DG, Brawley OW (2010). Effect of dutasteride on the risk of prostate cancer. N Engl J Med.

[30] Bhasin S, de Kretser DM, Baker HW (1994). Clinical review 64: Pathophysiology and natural history of male infertility. J Clin Endocrinol Metab.

[31] Gilligan T (2005). Social disparities and prostate cancer: mapping the gaps in our knowledge. Cancer Causes Control.

[32] Vidarsdottir H, Gunnarsdottir HK, Olafsdottir EJ (2008). Cancer risk by education in Iceland; a census-based cohort study. Acta Oncol.

[33] Cheng I, Witte JS, McClure LA (2009). Socioeconomic status and prostate cancer incidence and mortality rates among the diverse population of California. Cancer Causes Control.

